# Supplementary material for: Identification of a Plasma Microrna Signature as Biomarker of Subaneurysmal Aortic Dilation in Patients with High Cardiovascular Risk
Source: J Clin Med. 2020 Aug 28;9(9):2783. doi: 10.3390/jcm9092783 (PMC7565169; doi:10.3390/jcm9092783)
Supplement: Supplementary file 1 [file jcm-09-02783-s001.pdf]

Table S1. Demographic and clinical characteristics of patients with normal aortic diameter (CT), patients with ectatic aorta/subaneurysmal aortic dilations (SAD), and patients with aneurysmal aortic dilations (AAA) used for the screening experiment.

	**Pooled Patients**		
	**CT** (***n*** **= 6)**	**SAD** (***n*** **= 6)**	**AAA** (***n*** **= 6)**
Age, years	65 ± 3	67 ± 14	75 ± 6*
Male, *n* (%)	3 (50)	5 (83,3)*	4 (66.6)
Smoking habits, *n* (%)	1 (20.0)	4 (66.6)*	1 (20.0)
Alcohol, *n* (%)	1 (20.0)	4 (66.6)*	1 (20.0)
Hypertension, *n* (%)	6 (100)	6 (100)	6 (100)
Dyslipidemia, *n* (%)	4 (66.6)	4 (66.0)	2 (33.5)
T2DM, *n* (%)	2 (33.3)	3 (50)	2 (33.3)

Values are expressed as media ± SD for continuous variables or *n* (%) for categorical variables. *$p < 0.05$ vs. CT group. Abbreviations: T2DM: diabetes mellitus type 2.

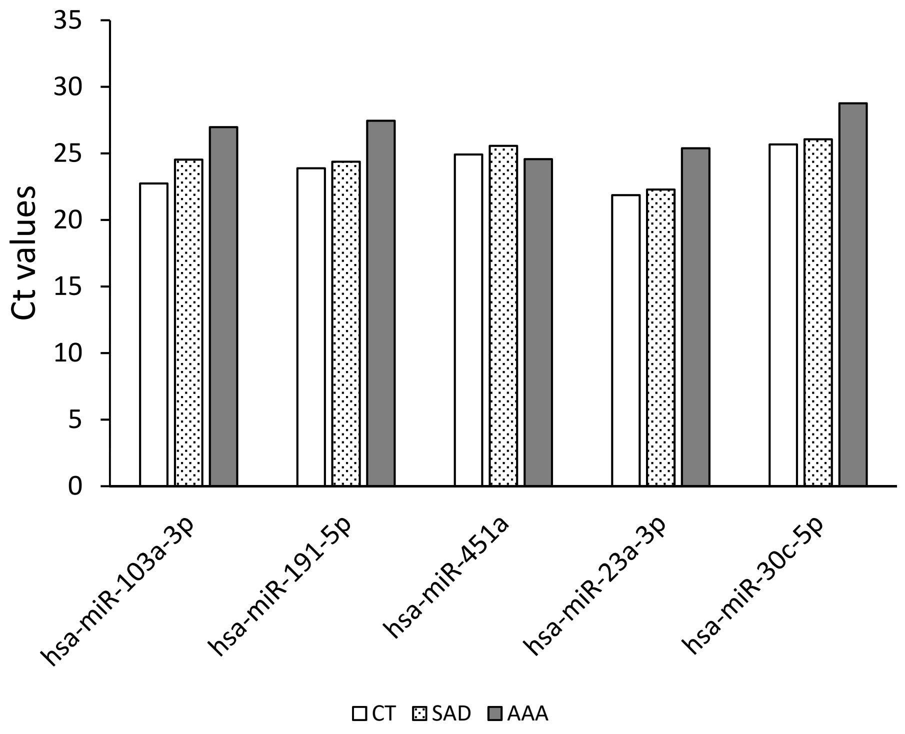

Figure S1. Expression levels of several miRNAS in pooled samples. We tested five candidates to reference gene, which were selected among genes that may be stably expressed in plasma/serum samples based on the literature or pre-existing data (Guidelines for the miRCURY LNA™ Universal RT microRNA PCR system, V2.0, Exiqon). MiR-451a showed to be good candidate to be a reference gene for normalization.
